# To die or not to die: a qualitative study of men’s suicidality in Norway

**DOI:** 10.1186/s12888-018-1843-3

**Published:** 2018-08-22

**Authors:** Birthe Loa Knizek, Heidi Hjelmeland

**Affiliations:** Department of Mental Health, Norwegian University of Science and Technology, NO-7491 Trondheim, Norway

**Keywords:** Suicidal acts, Men, Masculinity, Relationship problems, Attributed responsibility

## Abstract

**Background:**

Previous research has shown that men who adhere to traditional beliefs about masculinity have increased health risks compared to those who do not. Single marital status, unemployment, retirement, and physical illness are commonly known risk factors for male suicidal behavior. Most men struggling with these risk factors are, however, not suicidal. To find out more about what makes some men vulnerable to suicidal behavior, risk factors must be analyzed in light of men’s life history as well as the social context where they live their masculinity.

**Method:**

We conducted semi-structured qualitative in-depth interviews with 15 men (20–76 years old) who were admitted to hospital after a suicidal act. We analyzed the data by means of qualitative content analysis with a directed approach. The analysis was directed by the participants’ reports on whether they had wanted to die or not at the time of the suicidal act. On this basis, they were divided into two groups: a “to die” and a “not to die” group. We then analyzed each group separately before comparing them.

**Results:**

In both groups, the main reason or trigger for the suicidal act were problems in intimate relationships. These problems were complex and connected to the men’s lived masculinity, ranging from shame, or tainted masculine honor, to taking responsibility as a man for the wife. Some men pointed to pain and ennui as reasons or triggers for their suicidal act. Only one in the “not to die” group took full responsibility for the suicidal act, whereas all but one did the same in the “to die” group. The men not taking responsibility described the suicidal act as involuntary because of either alcohol or a kind of “black-out”. Not taking responsibility for the act may be a way of preserving masculine identity.

**Conclusion:**

Relationship problems are the main reason or trigger of the suicidal act for most participants, but in very different ways, mirroring lived masculinity. The most striking finding is the uniqueness of each story, questioning the utility of standardized suicide prevention efforts.

## Background

In 1998, Canetto and Sakinovsky [1] described the gender paradox of suicidal behavior: “In most Western countries, females have higher rates of suicidal ideation and behavior than males, yet mortality from suicide is typically lower for females than for males”. This paradox has puzzled suicide researchers for decades. Thus, gender has increasingly been emphasized as crucial for understanding suicidal behavior [2]. This is, for instance, reflected in the following comprehensive publications: In 2012, Canetto and Cleary [2] edited a part special issue of *Social Science & Medicine* on *Men, Masculinities and Suicidal Behavior.* In the same year, the Samaritans [3] in the UK published the research report *Men, Suicide and Society: Why Disadvantaged Men in Mid-Life Die by Suicide*. This report provided an extensive overview of factors leading to men’s suicide. In 2014, Lester, Gunn III and Quinnett [4] published the anthology *Suicide in Men: How Men Differ from Women in Expressing Their Distress*, with chapters from many different socio-cultural contexts. The editors’ aim with this book was to start a discussion on men’s vulnerability.

It is, however, necessary to be cautious since gender is one of the most frequently used sociodemographic variables as well as one of the most oversimplified and misused concepts in epidemiological and risk factor suicide studies [5–7]. Consequently, Krysinska et al. [5] underlined that “… it remains a serious challenge for both researchers and clinicians to identify risk and protective factors which make “some men” vulnerable to suicide, while others remain resilient when faced with life adversity or psychopathology”.

From research conducted to date, we know some of the circumstances that may make some men vulnerable to suicidal behavior. Evans et al. [8] and Scourfield & Evans [9] have, for instance, pointed out that men are challenged more by changing gender roles than are women. They also maintain that marriage may be a more positive experience for men than for women, that the care of children has become culturally more important and expected for men, and that men do not employ their social network for support when experiencing emotional difficulties. Hence, marriage breakdown may have worse consequences for men than for women.

Cleary [6] interviewed young Irish men after a suicide attempt and found that they had high levels of emotional pain. In addition, they had fewer coping skills as they had problems recognizing their symptoms as well as disclosing their pain to others because of dominating masculine norms of being in control. Also others have found men to be less likely than women to express emotions [10], which again has been assumed to account for the report of higher psychological distress among women, but higher suicide rates in men [6, 11]. A consequence of the inability to disclose emotional distress is that relationship problems and breakups might carry different weight for men than for women. However, the financial and ideological context of severe relationship problems must be considered as mediating factors [8, 12, 13] as cultural condemnation of divorce or women’s financial dependency influence the effect of relationship breakup. In addition, one has to consider when in the life course the breakup happens as it carries different weight during different age groups. According to a systematic review by Evans et al. [14] middle-aged men appear to be more affected than women and younger men by a breakdown in their marital/romantic relationships since these relationships may be their main source of intimacy and their only possibility for sharing their vulnerable sides. In another review study Evans et al. [8] find “… no definitive evidence of a gender differential in suicidal behaviors following the breakdown of a relationship” as they do not find any clear trend that goes across age. Nor do they find consistent patterns across countries or regions.

Previous research has shown that men who adhere to traditional beliefs about masculinity seem to have increased health risks compared to those who do not aspire to traditional forms of masculinity [15]. This must be seen in relation to the fact that traditional masculine behavior underplays the role of emotion as the man is expected to be in total control of himself and the situation, and thus reluctant to report distress [16]. This, in turn, then allows stress to build up and a vulnerability to suicidal behavior may develop. However, the development of masculinity occurs in interplay with the actual and constantly changing context. The life of an 80-year old man has developed in a considerably different context than what is the case for a 20-year old man. From the existing research, some male risk factors like single marital status, unemployment, retirement and physical illness are identified [17], but they all must be seen in light of the actual social and normative context, the specific life history and age and what they mean for each man.

Historically, masculinity norms in Norway were influenced by harsh physical environment and being a fishing nation [18]. Dangerous and hard work was fundamental for being able to provide for the family and necessary periods of absence built the fundament for the women to take responsibility. According to Lease et al. [18] this created cultural values of self-sufficiency, independence, and courage of men, but lay also the foundation for egalitarianism between the genders. Men supported changes at home and working-place but they may have received incongruent messages about appropriate masculine behavior. The news featured Norwegian men who felt a devaluation of traditional masculine roles [19]. In addition, the Norwegian society is changing rapidly through immigration and masculine norms from other cultures are blended into society.

In a changing society like Norway, context sensitive research is needed and qualitative research comes to the fore. Such research is able to take more of the context and actual life situation of individuals into consideration in the analysis, compared to what quantitative risk factor research is able to [8, 20]. Qualitative research also allows us to focus on the individual and to highlight contextualized individual differences in circumstances related to suicidality. This is important in order to develop our understanding of what suicidality might mean to people who are suicidal, in their context, and beyond the common simplistic risk factor categories. Franklin et al.’s [21] recent meta-analysis of 50 years of risk factor research demonstrated the limited value of risk factors in terms of understanding suicidality and hence for suicide prevention. For example, “relationship problems” is a commonly found risk factor for suicidal behavior. In her comprehensive review study on Muslim women and suicide, Canetto [22], however, found that relationship problems can cover many, and very different issues for these women. There is no reason to believe that this would be any different for women in general, as well as for men. This will in turn have consequences for suicide prevention. The purpose of the present qualitative interview study was thus to investigate what men who have engaged in a suicidal act perceived as crucial for their decision to harm themselves or attempt to take their life. We analyze this in the context of their actual life situation, and hence look deeper into aspects that might be overlooked or not described in traditional quantitative risk factor research.

## Method

We conducted semi-structured qualitative in-depth interviews with men admitted to hospital following a suicidal act. Before the interview, we knew nothing about the participants other than the fact that they had harmed themselves sufficiently to require hospitalization. We chose this approach deliberately in order to be as open as possible to the men’s own descriptions of the circumstances around their suicidal act.

### Participants

We interviewed 15 men (20–76 years old), recruited through the hospital. Six of the men were in a stable relationship, five were currently going through, or had just been through a divorce, whereas the rest were single. Five had a job, six were unemployed, two were retired, and two were on sick leave and/or underwent re-education. Two of the men had higher education. Four lived alone, whereas the others lived with a wife, a male/female lover, a child, or a friend. Only one of the men mentioned that he had some mental disorder diagnoses, namely anxiety and depression, and that he was on psychopharmacological medication. Some others said they had been offered antidepressants after incidents/accidents, after which they had felt depressed. Two of the men revealed having been sexually abused from childhood. As regards methods used in their suicidal acts, 13 had taken an overdose of medication, one had swallowed a diluent, and one had tried to hang himself. Alcohol was involved in five of the suicidal acts, whereas only two claimed to have problems with alcohol in general, and two others admitted taking drugs. Almost two thirds (*n* = 9) reported sleeping problems.

### Procedure

The second author interviewed the participants one to four weeks after their suicidal acts. The semi-structured interview guide was composed of a narrative part and a problem-focused part. In the narrative part, the men were requested to describe the circumstances leading to their suicidal act, the suicidal act itself, as well as what reactions they had been met with after the act. In the problem-focused part, the interviewer asked questions related to their health if this was not sufficiently covered in the narrative part. The interviews lasted between 19 and 160 min, with most of them lasting about one hour. The interviews were recorded and transcribed verbatim.

### Data analysis

We analyzed the data by means of qualitative content analysis [23] following a directed approach [24]. Berelson, 1952, developed content analysis originally as an exclusively quantitative approach. Since then it has undergone comprehensive changes and has moved into the qualitative realm and interpretative perspective [25]. In contrast to context analysis in the positivistic paradigm, here the assumption is that data and interpretation are co-creations of interviewer and participant. In the analysis the co-creation is between the researchers and the text. In our approach we have combined an inductive and a deductive approach. In the initial inductive analysis, the entire material was read by the two authors and the observation of a difference between the men who wanted to die and those who did not want to, triggered a more deductive approach looking at differences between those two groups in the further analysis. Our analysis became more directed [24], which implies coding of the text directed by theory or research findings. In our case, the analysis was directed by the participants’ reports on whether they had wanted to die or not at the time of the suicidal act. Two thirds (*n* = 10) explicitly stated that they had wanted to die, whereas one third (*n* = 5) said they had not wanted to die. Consequently, we divided the sample into two groups: a “to die” group and a “not to die” group. In the further process, we stayed close to the text and coded it in line with phenomenological guidelines and developed categories, which were further developed into themes. We then compared the two groups of men in relationship to these themes. This flexible analytical approach has been described as the researcher taking various scientific positions depending on the aim of the study [25]. The aim of this study was thus to investigate what men who had engaged in a suicidal act perceived as crucial for their decision to harm themselves or attempt to take their life and analyze this in the context of their actual life situation.

### Ethical considerations

The Regional Committee for Medical and Health Research Ethics approved the study. All the participants had given informed consent before the interview. All interviews took place in an office at the outpatient clinic that was responsible for the participants’ follow-up after their suicidal act. The participants were offered a follow-up by the staff at the outpatient clinic after the interview. None of the participants needed such follow-up.

A main purpose of the present paper was to highlight some contextualized individual differences regarding what the men perceived as crucial for their decision to harm themselves or attempt to take their life. This has the potential to challenge the principle of anonymity, which we have handled in the following ways. With the quotations, we report age groups rather than exact age: young adults, middle-aged, and elderly, here defined as 20–35 years, 36–60 years, and 60–76 years, respectively. We have also changed some potentially identifying features of the participants of whom we present rather detailed descriptions and quotations (features presumed to be unimportant for understanding the suicidality).

## Results

Two themes were developed through the analysis: *Reasons or triggers for the suicidal act* and *Attributed responsibility for the suicidal act.* They are described and substantiated by quotations in the following.

### Perceived reasons or triggers of the suicidal act

All the men in the “not to die” group reported problems with or loss of partner/wife as main triggering factor for their suicidal act. Even though they admitted that problems had accumulated for some time, they pointed to the relational problem as the last straw that broke the camel’s back: “It was her that triggered it. And I mean it, that it was her that triggered that this happened. The way that she made me look like a fool in that situation” (middle-aged man). The situation that triggered his suicidal act was that his partner in the presence of friends tried to change some plans they had made as a couple, and instead wanted to plan things that included the friends. His narrative pointed at the situation rather than the partner as the trigger, even though his way of expression was equivocal. He even repeatedly expressed sympathy with his partner’s situation after his suicidal act. In the situation he felt let down, excluded, and ridiculed, and then harmed himself. He felt he had lost face in the situation and could not bear the shame. This man was the only one in the “not to die” group who still lived together with his partner (at the time of the interview).

All the other men in the “not to die” group were separated or divorced, and reported feeling lonely or let down. One found it problematic to describe a reason or trigger for his self-harm, but finally ended up saying: “But I went through a separation, which was pretty hard and was much unexpected” (middle-aged man). Before the separation, his parents-in-law had promised to support his business financially on the condition that his wife divorced him. He refused to separate, but his wife divorced him anyway and he did not receive any financial support. He consequently ended up alone with financial problems, felt deceived by his in-laws, and abandoned by his wife. Another middle-aged man had led a life dominated by alcohol and illness that ended up with impotence and a separation from his wife. One young man met a previous girlfriend, who had dumped him earlier, as a pregnant woman in a new relationship. Although the circumstances of these two latter men were quite different, both struggled with loneliness and the loss of relationship and were unable to accept the separation.

As the men illustrated above, a breakdown in relationships involved many different issues, such as impotence, loneliness, betrayal, abandonment, and shame.

Half of the larger “to-die” group also reported relationship problems as main reason or trigger for their suicidal act, and again this covered very different issues. Of the 10 men, four lived alone and three of these four were currently undergoing separation/divorce. One of the other five still lived with his parents, whereas the rest lived in stable relationships. Two of the men still living with their partner mentioned infidelity or suspicion of infidelity as a triggering factor. One man reported having been institutionalized and helpless when his wife took all their valuables and went away with another man. His helplessness deepened the feeling of being betrayed by his wife. He describes himself as wanting to be independent and able to manage his problems by himself, but admits that there is a limit to what one can take. Another participant was the victim of rumors regarding his wife’s infidelity; rumors that eventually turned out to be false. Both reported perceived infidelity as the factor that triggered their suicide attempt, although the circumstances were very different.

Three men mentioned illness/pain and, not surprisingly, the two oldest participants were among them. The third was a young man with serious health problems. All three experienced no quality of life and were without hope for improvement of their condition. The two elderly men expressed explicitly that they did not want to be a burden. They seem to adhere to the traditional masculine values of independence and autonomy, and for one of them death became almost a practical solution in order to take care of the wife: “And have talked about it [with wife] that maybe it is easier for you if I was gone. You could just have sold everything and then bought a flat, easy” (elderly man). He was worried that his wife was overwhelmed by the workload since he could not contribute the way he wanted anymore, and she had to take care of him in addition. At the same time, he insisted that he was *not* depressed, because he did not see himself as the “depressive type”. Another elderly participant also suffered from severe illness, which would increasingly disable him and make him dependent on others. He described himself as a modest person, who did not like to ask for continuous assistance and as a consequence he could not bear the thought of the future based on dependency.

The younger man who mentioned pain as a trigger for his suicide attempt had completely lost hope in the health care system, whose lack of ability to understand and help he found scandalous:

“I just get more and more sick and become a larger and larger problem for the health care system (…) and I feel totally ignored and overlooked. Everything that has been found out I have found out myself.” (…) You know, I know a lot about bodily processes and then, then the doctors get grumpy (…) Then you get pushed like a thing, well, like a hot potato, which nobody wants to hold in his hands (young man).

The pain and the treatment from the health care system seemed too much and he attempted suicide twice. He felt that there was no hope left and that life was nothing but pain.

The last two men reported being tired of life as the reason for their suicide attempt. Their life situation and history were, however, very different. One was a young man living with his parents, with whom he felt he had nothing in common. He claimed to have no friends, no interests and no energy and insisted that he would not change. In his opinion, he did not fit into his family or into the world as such. Without a prospect of change, he had no hope for the future:

“I tell you that I will take my life regardless what happens in the future and I will do it as soon as … .well, as soon as possible. And that is nothing people can do anything about. (…) The way I see it is that if you are going to live, you must have something to live for or at least something to look forward to, and that I have never had and will never get. So I see no reason why I should stay here then” (young man).

He lived in a social vacuum and without interests or energy to make a change, he felt tired of life. He was the only participant reporting having been diagnosed with any mental disorders (depression and anxiety).

The other participant being tired of life was middle-aged and had lived an active life with a lot of interests and engagements. Despite a turbulent upbringing, he managed to get an education and a family. He had been politically engaged and had strong ideals for how the world should be. Now his children had grown up, he and his wife had divorced amicably, and he was not happy with the changes in the world. He felt that the world had become cold and cynical: “I don’t like the world we live in today (…) I don’t feel at home in the world, we live in” (middle-aged man). For the last 10 years, he had not wanted to live anymore and felt burned out. He had attempted suicide before, but in the last moments of consciousness, “a spark of life” emerged and then he got ambivalent. He underlined that it is not necessary to be depressed for not wanting to live in the world as it looks now. He saw his decision as a conscious choice.

In summing up the reasons or triggers, the men mentioned for their suicide attempts, it is evident that their stories are *very* different. Even when it is possible to categorize them as, for instance, having relationship problems or physical pain, the differences in these categories seem larger than the similarities.

### Attributed responsibility for the suicidal act

Most of the men explicitly allocated responsibility for their suicidality. In the “not-to-die” group, only one man felt entirely responsible for the suicidal act himself. He was, however, ambivalent as he found the telephone number for the emergency services before harming himself. The others in this group insisted on not having been in possession of their faculties, for instance, because of alcohol:

“I have sleeping problems during night or rather always. Thus, I got sleeping pills from my doctor … and …. I took all of them at once. Ninety pills. And that was after a large consumption of alcohol, so … I don’t think I knew that I took them” (middle-aged man).

One of the other participants in this group only drinks occasionally, while another one admitted having problems with “King Alcohol”, which makes him crazy. Another man mentioned that the brain had “clicked” in the moment of the suicidal act:

It did not hurt. Because I was gone. I was - it was not me who did this. It was not I, who took the rope in the staircase and put it up. It was not me. It was a different side of me, it was a “click” up in my brain, which made it happen. As simple and easy as that (middle-aged man).

He distanced himself entirely from the act. This made it possible for him to sympathize with the reactions of his partner and even to admit that the purpose of the act probably had been to scare his partner, without displaying any guilt. He emphasized that his case shows that suicidality can happen to “completely normal people”: “It was not I who was that person obviously, because I remember nothing.”

The picture in the “not-to-die” group differed significantly from the one in the “to-die” group; only one in the latter group was not ready to take responsibility for the suicidal act, whereas the rest took full responsibility. The youngest one of the participants, who was tired of life, distanced himself completely from the act and underlined that he did not do it of his own free will:

“I would never have a serious suicide attempt, because an attempt is not what I stand for. I mean that if you have to do it, you do it. And therefore I think it is a bit embarrassing what happened, because that, that was a thing that goes against everything I stand for. But as I said, I would never have done it if I had been conscious of what I was doing. So, I don’t know what happened or why it happened, but it is probably some blackout, as they have called it. But I shall not discuss that” (young man).

He felt his honor tainted, because if he had been in possession of his full faculties, he would have been dead. The suicidal act was for him an embarrassing failure and he was ashamed because of it. He ascribed the failure to a blackout.

Some of the other men in the “to-die” group reported having thought about it a long time in advance, while others spent some time contemplating about it in the course of the suicidal act, for example: “… .and then it was, it was totally calm, it was precisely as it should be when I had done it. It was entirely appropriate” (middle-aged man). This participant lost hope when he lost both partner and job and as a result felt worthless and unwanted. He did not regret the attempt at that moment and seemed content with his fate and he emphasized that a theme in his life was to think that he probably did not deserve anything better.

For all men in the “to-die” group, suicide seemed to be a deliberate choice and the best option in their situation. The men reported that they were sure they wanted to die, but for three of them ambivalence came into play when the pain became intolerable or right after the attempt, and they called for help themselves:

“When I did it, I was very clear; very conscious. But then I lost courage (…) So … a part of you will die and a part of you will live and then you become very ambivalent and then you telephone and get things going. So, it is really about getting over the threshold that you are not afraid anymore: this time I make it” (middle-aged man).

The challenge for this middle-aged man was to get beyond the fear of death. Like the other two men who had second thoughts, he seemed unable to carry out his wish/intent to take his life. However, this inability appears in different shapes: whereas one had the telephone number of the emergency department ready before the suicidal act, the two others found it and called after the act. For one of them, it was the first time he had attempted suicide and he seemed ambivalent. For the other, it was his third suicide attempt and he called because he, in his own words, lost the courage after having swallowed sleeping pills. Another participant could not bear the pain after having drunk a diluent, which had also been the problem with his previous (first) suicide attempt. Part of some of the stories was a regret at having disrupted the suicide attempt.

## Discussion

In this study, we interviewed men after a suicidal act medically serious enough to require hospitalization. One-third of the men expressed that they actually had not wanted to die, while the rest had decided that death was the best option in their life situation. We compared these two groups on the two themes developed in the analysis, namely what they perceived as the main reason or trigger for their suicidal act and to whom/what they attributed responsibility for the act. All the men who did not want to die and half of the men who had wanted to die emphasized relationship problems as the main reason/trigger for the suicidal act. The elevated risk of suicidal behavior and ideation after breakdowns in intimate relationships has been known for some time [26]. Krysinska [5] has emphasized a male vulnerability to negative life events, while Lester, Gunn and Quinett [4] emphasized men’s vulnerability specifically to relationship problems: “Men also appear to be more impacted by breakdowns in their marital and romantic relationships, perhaps because they benefit more from marriage and perhaps because men find it hard to meet the modern expectations for increased intimacy”. In their systematic review of research on relationship breakdown and suicide risk, Evans et al. [8, 14] found 17 studies with higher risk for men, six with a higher risk for women, and six with no gender difference. Van Orden et al. [27] pointed out that women in general value family and being loved higher than men and then in the absence of these “… suffer greater emotional pain than do men in the same situations”. This stands in contrast to Evans et al. [8, 9], who underline men’s larger vulnerability under relationship breakups and suggest as explanations “… men’s role inflexibility, the increasing importance of the care of the children, men’s desire for control in relationships, and men’s social network” [9].

In order to understand and target this vulnerability we need to know more about particularities, as we meet different reactions to problems based on men’s masculine identity. Three quarters of our participants described relationship problems as the main reason or trigger for their suicidal act, though they were related to different underlying feelings such as feeling betrayed, abandoned, ashamed, or being a failure in terms of inability to take responsibility for the wife. The individual emotional reactions must be understood on the background of their history and context and their relationship with those. Several publications and anthologies [2, 4, 28] have contributed significantly to the contextualized understanding of gendered suicide scripts, while suicide among men in the specific Norwegian context still is mainly unexplored. With the exception of Rasmussen et al. [29], who have contributed with their psychological autopsy study on suicide among young men, where they found suicides to be signature acts of compensatory masculinity, we still do not know much about the particularities among men in Norway.

In the present study, the main trigger for the suicidal act among our participants was in line with other countries a variety of problems related to close relationships. In fact, three quarters of them mentioned such problems that covered many different issues and the men also felt hurt in different ways as their actual background and context was different. In addition, the age range was from 20 to 76 years, contributing even more to the complexity of the situation as their situation and psychological make-up might have differed significantly. The relationship problems mentioned were connected to the men’s lived masculinity, ranging from shame, or tainted masculine honor, to taking responsibility as a man for the wife. Most of these problems could be interpreted in line with men’s desire for control in relationships as a consequence of the traditional Norwegian masculinity norms as independence and self-sufficiency. The two oldest men stated that they had not wanted to be a burden to their wives, which could be interpreted as role inflexibility [9]. In 2017, Canetto, in her article “Suicide: Why are older men so vulnerable?” [30] convincingly showed that illness may lead men to suicide in a much higher degree than women. Her explanation is “Rigidity in coping and in sense of self, consistent with hegemonic-masculinity scripts, emerged as individual-level clues”. The meanings and consequences of severe illness thus are highly dependent on gender values and the ideal of self-sufficiency and independence that traditionally were Norwegian masculine ideals. Except for this perceived burdensomeness, we could not find any trigger specific for the age groups (young adults, middle-aged, old).

Relationship problems and perceived burdensomeness draw the attention towards the *Interpersonal Theory of Suicide* (*IPTS*); a theory developed by Joiner [31] and further explicated by Van Orden and colleagues [27]. Judging from the recent systematic review and meta-analysis of the studies testing this theory to date conducted by Chu and colleagues [32], this theory currently seems to dominate the suicide research field. The *IPTS* posits that thwarted belongingness and perceived burdensomeness must be present *simultaneously* for a suicidal desire to emerge.

Certainly, some of the (relationship) problems found above may be interpreted as thwarted belongingness, but this does not seem to be accompanied by feelings of burdensomeness. The men who explicitly said that they felt like a burden (to their wives), expressed this in a context of long lasting, stable relationships, indicating that this had nothing to do with thwarted belongingness. In his book outlining the *IPTS*, Joiner [31] claims that this theory is able to explain suicidality “worldwide, across cultures” and in their meta-analysis of all the 122 studies testing the *IPTS* to date (all quantitative), Chu et al. [32] on the one hand claim that the findings support the theory, but on the other admit that the evidence is mixed, and that effect-sizes are small to medium, indicating huge variations. Qualitative studies like the present one, are able to present more nuanced and contextualized pictures of what lies behind suicidal behavior compared to what quantitative risk factor research is able to. In qualitative studies, some of the complexity and individual variability involved in suicidal behavior becomes apparent [20]. Such studies contribute to question the relevance of reductionist theories like the *IPTS,* which posits that suicide can be explained by three factors only (acquired capability to enact lethal self-harm in addition to thwarted belongingness and perceived burdensomeness). However, (masculine) identity always is contextual and dynamic, developed and maintained in an ongoing interplay with other people and the surrounding society. Consequently, the prominent approach to suicide prevention seems to require a dynamic and systemic perspective instead of a pure individualistic approach.

In keeping with Fleischer [33] and Staples & Widger [34], we found suicidality to be clearly relational. During the last 60 years this relational aspect of suicidality repeatedly has been described in communicative terms as ‘symbol’ [35], ‘appeal’ [36], ‘manipulation’ [37] or ‘cry for help’ [38], and hence defined suicide as a communicative act. Men’s hesitance to express distress and seek help might be an additional argument to study their suicidal behavior as their specific form for communication in order to understand why it seemed necessary to turn from verbal communication to suicidal acts. It might therefore be fruitful to view suicidal acts as relational, communicative acts and thus to interpret them within the framework of communication theory [33, 39].

In this study we found an interesting difference in attribution of responsibility between the “not to die” and “to die” group. Only one in the “not to die” group took full responsibility for the suicidal act, whereas all but one did the same in the “to die” group. The men who did not want to die described the suicidal act as involuntary because of either alcohol or blackout. This attribution of responsibility goes beyond classical attribution theory [40], as the men who renounced their own responsibility for the suicidal act, did not attribute it externally to someone else, but to forces that were beyond their power within themselves. For three men, the explanations seemed to offer a way of distancing themselves from the act without feelings of guilt or shame. This mechanism enabled them to sympathize with those close to them and create a shared project to deal with the problems. The other two participants who did not take responsibility, felt betrayed by their family and did not want to talk with them, and only reluctantly with health personnel. The denial thus did not create a constructive platform for these men. The only man in the “not to die” group taking full responsibility for his suicidal act was ashamed, did not want to talk to either family or professionals and just wanted to “pull himself together”.

In the “to die” group, all but one took full responsibility and the one not doing so described the suicidal act as a “failure”, as he was determined to die. His ashamed reaction to the ‘failed’ attempt can be understood in line with Canetto and others’ work on gendered cultural scripts of suicidal behavior [2, 4], as a failure of the masculine ideal of being in full control.

The differences in attribution of responsibility might indicate the level of determination or a way of ascribing meaning to the suicidal act at the same time as maintaining their masculinity. They described the suicidal act as a deliberate act; an act they had thought extensively about for a longer or shorter period of time. An important question might be whether their attribution of responsibility and thus level of determination and meaning-making might be an indicator of the prognosis for their future and their proneness to repeat the suicidal act, with or without lethal intent. This has implications for suicide prevention.

The diversity of the stories speaks heavily against standardized efforts, including categorizing patients in relation to the level of suicide risk. The time should rather be spent listening to patients’ stories. This is in line with comprehensive research, which shows that clinicians should stop categorizing patients in relation to the level of suicide risk, and that health authorities should withdraw guidelines that require this [41]. The diversity of stories and needs of the patients rather calls for time to build up strong meeting-points with health personnel [42, 43]. Professionals must be given space, time and trust to apply their health professional skills when meeting the individual patient [44]. This requires the health professionals to be provided with suicidological competence that far exceeds a biomedical understanding of suicidality [45]. Not taking responsibility for a suicidal act might for example be an important message for health professionals to explore why the men renounce responsibility and which psychological function this may have. This should be addressed in the follow-up of suicide attempts by men and should also be studied in depth in further context sensitive research.

## Conclusion

Relationship problems were the main reason or trigger of the suicidal act for most participants, but in very different ways, mirroring lived masculinity. As relationship problems cover a vast variety of issues that are irrefutably intertwined with their specific history and context, standardized efforts in terms of suicide prevention might not be the way forward. Besides a variety of relationship problems, some men also described ennui and physical pain as main reason/trigger of the suicidal act. The most striking finding of our study, however, is the uniqueness of each story.
